# Adrenomedullin Regulates* IL-1β* Gene Expression in F4/80+ Macrophages during Synovial Inflammation

**DOI:** 10.1155/2017/9832430

**Published:** 2017-02-19

**Authors:** Shotaro Takano, Kentaro Uchida, Masayuki Miyagi, Gen Inoue, Jun Aikawa, Kazuya Iwabuchi, Masashi Takaso

**Affiliations:** ^1^Department of Orthopedic Surgery, Kitasato University School of Medicine, 1-15-1 Minami-ku Kitasato, Sagamihara City, Kanagawa 252-0374, Japan; ^2^Department of Immunology, Kitasato University School of Medicine, 1-15-1 Minami-ku Kitasato, Sagamihara City, Kanagawa 252-0374, Japan

## Abstract

Adrenomedullin (AM) plays an important role in the regulation of inflammatory processes; however, the role and expression of AM in synovial inflammation have not been determined. To investigate the expression and role of AM in inflamed synovial tissue (ST), the gene expression profiles of AM in the ST, including synovial macrophages and fibroblasts, of a murine patellar surgical dislocation model were characterized. In addition, the effects of interleukin-* (IL-) 1β* and AM in cultured synovial cells were also examined. CD11c^+^ macrophages were found to be elevated in ST of the surgically dislocated patella. Higher gene expression of* CD11c*,* IL-1β*,* AM*, receptor activity-modifying proteins 2* (RAMP2)*, and 3* (RAMP3) *was also observed in ST obtained from the dislocated side.* AM *expression was also significantly increased in synovial fibroblasts and macrophages in response to IL-1*β* treatment. Synovial macrophages also highly expressed* RAMP3 *compared to fibroblasts and this expression was further stimulated by exogenously added IL-1*β*. Further, the treatment of the F4/80-positive cell fraction obtained from ST with AM inhibited* IL-1β* expression. Taken together, these findings demonstrated that AM was produced by synovial fibroblasts and macrophages in inflamed ST and that increased levels of AM may exert anti-inflammatory effects on synovial macrophages.

## 1. Introduction

The synovial membrane lines the cavity of synovial joints and is composed of macrophage- and fibroblast-like synoviocytes and an underlying layer of synovial tissue. Synovial macrophages produce several inflammatory cytokines, including interleukin- (IL-) 1*β*, IL-6, and tumor necrosis factor- (TNF-) *α*, which contribute to arthritis progression and associated joint pain [1–3]. For this reason, the regulation factor of these cytokines in synovial tissue (ST) may aid in the understanding of arthritis pathogenesis and synovial inflammation.

Adrenomedullin (AM) is a 52-amino-acid vasodilator peptide that is released from vascular smooth muscle and endothelial cells during inflammation and plays an important role in the regulation of inflammatory processes [4–9]. AM belongs to the calcitonin gene-related peptide (CGRP) family and was originally identified in human pheochromocytoma tissue using elevated platelet cAMP activity as an indicator [6]. AM induces its biological effects by modifying the activity of the calcitonin receptor-like receptor/receptor activity-modifying protein 2 or 3 receptor complex [10, 11]. Plasma AM concentrations are reportedly elevated in rheumatic disorders [12, 13], and exogenously administered AM has anti-inflammatory effects on murine monocyte/macrophage cell lines [14] and lung inflammation models [15]. To date, however, the role and expression of AM in synovial inflammation have not been determined.

To determine if AM regulates the function of synovial macrophages during synovial inflammation, here, we characterized the expression profiles of AM in the inflamed synovial tissue of mice. In addition, the regulation of AM expression by inflammatory cytokines and effects of AM on synovial macrophages and fibroblasts were also examined.

## 2. Materials and Methods

### 2.1. Animals

Specific-pathogen free colonies of 9-week-old C57BL/6J mice were maintained at Nippon Charles River Laboratories (Kanagawa, Japan). The mice were housed throughout the study in a semibarrier system under a controlled environment (23 ± 2°C; 55%  ± 10% humidity; 12 h light/dark cycle). All of the experimental protocols were approved by the Kitasato University School of Medicine Animal Care Committee.

### 2.2. Induction of Synovial Inflammation

Synovial hyperplasia was induced in mice using a surgical technique. Briefly, mice were anesthetized, and a medial parapatellar arthrotomy was performed on one knee (dislocation side) by first extending the joint and then dislocating the patella laterally. The joint was then fully flexed, and the rectus femoris was sutured on the lateral side, followed by suturing of the skin. The contralateral knee was not subjected to surgery as a control.

### 2.3. Flow Cytometric Analysis of ST Cells

One week after surgery, C57BL/6J mice were sacrificed by the intramuscular injection of a mixture of medetomidine, midazolam, and butorphanol tartrate. Skin was removed with a scalpel, and ST was harvested and then digested with 1 mg/mL type I collagenase for 2 h at 37°C. The released cells were reacted with antibodies against F4/80 (clone: BM8), CD11b (clone: M1/70), and CD11c (clone: N418) (Biolegend, CA, USA), and staining with 7-amino actinomycin D (7-AAD) was used to identify dead cells. Isotype-matched antibody controls were also used as a negative control. For each synovial cell sample, 30,000 live, single-cell events were analyzed by flow cytometry (FACSVerse; Becton-Dickinson).

### 2.4. Isolation of F4/80-Positive Cells from ST

ST-derived mononuclear cells were isolated by the digestion of excised ST with type I collagenase for 2 h at 37°C and were then suspended in 500 *μ*L phosphate-buffered saline (PBS) containing biotinylated anti-F4/80 antibody. After incubation for 30 min at 4°C, the cells were washed with PBS and mixed with streptavidin-labelled magnetic particles (BD IMag Streptavidin Particles Plus-DM; BD Biosciences, Tokyo, Japan), and labelled cells were subjected to magnetic separation using an IMag separation system (BD Biosciences). After 30 min incubation on ice, unbound (F4/80-negative) cells (fibroblast-rich fraction) were collected by the addition of warmed (37°C) *α*-minimum essential culture medium (MEM) to the cell suspension. The tube containing the bound cells was removed from the magnetic support and F4/80-positive cells (macrophage-rich fraction) were collected by the addition of 3 mL *α*-MEM. The F4/80-positive and F4/80-negative cell fractions were centrifuged at 300*g* for 10 min and the obtained cell pellets were cultured in six-well plates containing *α*-MEM. The cellular gene expression of* CD11c*,* IL-1β*,* AM*, and* RAMP3* was analyzed by reverse transcription-polymerase chain reaction (RT-PCR). The cell isolation and expression analysis was performed in five times.

### 2.5. Effect of IL-1*β* and AM on ST-Derived Cells

Collected F4/80-positive and F4/80-negative cells from C57BL/6J mice were cultured in *α*-MEM in six-well plates for 1 week at 37°C in a 5% CO_2_ incubator. Synovial fibroblasts and macrophages were then incubated with 0 (control group), 5, or 50 ng/mL mouse recombinant IL-1*β* (Biolegend, San Diego, CA, USA) and were also stimulated with 0 (control group), 10^−7^, and 10^−6^ M AM (Phoenix Pharmaceuticals, USA) for 24 h.* IL-1β*,* AM*,* RAMP2*, and* RAMP3* expression was analyzed by RT-PCR. The experiment was performed four times.

### 2.6. Real-Time Polymerase Chain Reaction (PCR)

Total RNA was obtained from ST and cultured F4/80-positive (macrophage) and F4/80-negative (fibroblast) cells using TRIzol reagent (Invitrogen, Carlsbad, CA, USA) according to the manufacturer's instructions and was used as template for first-strand cDNA PCR synthesis. The PCR reactions were performed with reaction mixtures consisting of 2 *μ*L cDNA, a specific primer set (0.2 *μ*M final concentration), and 12.5 *μ*L SYBR Premix Ex Taq (Takara, Kyoto, Japan) in a final volume of 25 *μ*L. The primers were obtained from Hokkaido System Science Co., Ltd. (Sapporo, Japan) and were designed using Primer Blast software (Table 1). The specificity of the amplified products was confirmed by melt curve analysis. Quantitative PCR was performed using a real-time PCR detection system (CFX-96; Bio-Rad, Hercules, CA, USA) and the following cycling parameters: initial denaturation at 95°C for 1 min, followed by 40 cycles of 95°C for 5 s, and 60°C for 30 s. The level of target mRNA expression was normalized to that of glyceraldehyde-3-phosphate dehydrogenase (GAPDH).

### 2.7. Statistical Analysis

The paired *t*-test was used to examine differences in the ST of control and dislocated patella. The paired *t*-test was also used to examine differences between F4/80+ and F4/80− cell fractions. One-way ANOVA with Fisher's least significant difference (LSD) test was used to examine differences among the control group and the group stimulated with either low or high concentration IL-1*β* or AM. All statistical analyses were performed using SPSS software version 19.0 (SPSS, Inc., Chicago, IL, USA). A *P* value of < 0.05 was considered statistically significant.

## 3. Results

### 3.1. Characterization of Macrophage Populations in ST

**Figure 1:**
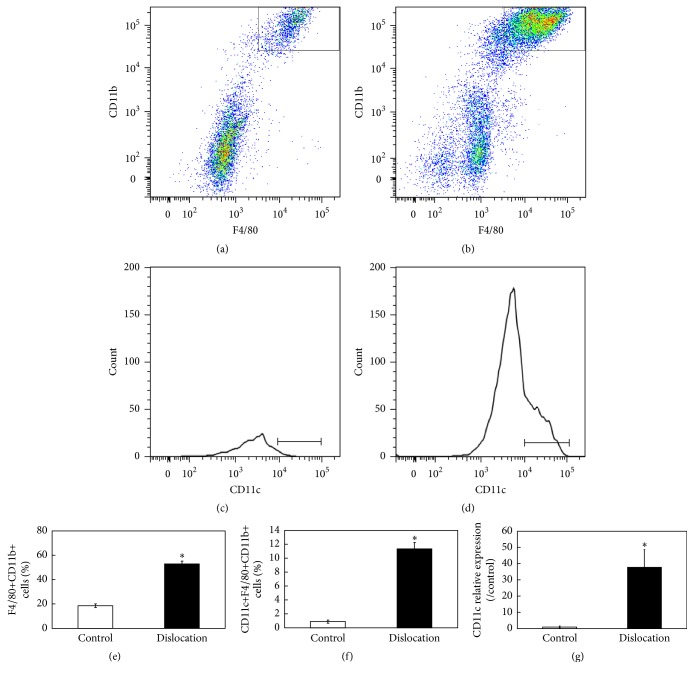
Characterization of macrophage cells in inflamed synovial tissue of C57BL/6J mice. Flow cytometric analysis of CD11c^+^F4/80^+^CD11b macrophage cells in the synovial tissue (ST) obtained from the control and dislocated side of a patellar surgical dislocation model generated in C57BL/6J mice (a–f). (a, b) Dot-plot analysis of F4/80^+^CD11b^+^ cells in ST of the control (a) and dislocated side (b) of mice; *x*-axis, F4/80; *y*-axis, CD11b. (c, d) Histogram analysis of CD11c^+^ cells in the gated regions in the dot-plots of ST cells isolated from the control (c) and dislocated side (d) of mice. Percentages of F4/80− and CD11b^+^ cells (e) and CD11c^+^ cells in the F4/80− and CD11b-positive gated regions in the ST of control and dislocated side (f) of mice (*n* = 5). (g) Real-time PCR analysis of CD11c expression in the ST obtained from the control and dislocated side of C57BL/6J mice (*n* = 5). ^*∗*^Statistically significant difference between the control and dislocated side. All data are presented as the mean ± standard error (*n* = 5).

Proinflammatory macrophages in synovium express CD11c [16, 17]. Here, the proportion of CD11c^+^ macrophages in the ST of a murine patellar surgical dislocation model was investigated by flow cytometry. The number of F4/80^+^ CD11b^+^ (Figures 1(a), 1(b), and 1(e)) and CD11c^+^ F4/80^+^ CD11b^+^ macrophages (Figures 1(c), 1(d), and 1(f)) in the ST of the dislocated patella was significantly higher compared to that found in the ST of the contralateral (control) side. Consistent with this finding, CD11c mRNA expression in the ST of the dislocated side was significantly higher compared to that detected in the ST of the control side (Figure 1(g)).

### 3.2. Expression of the* F4/80*,* CD11c*,* IL-1β*,* AM*, and* RAMP3* Genes in ST

**Figure 2:**
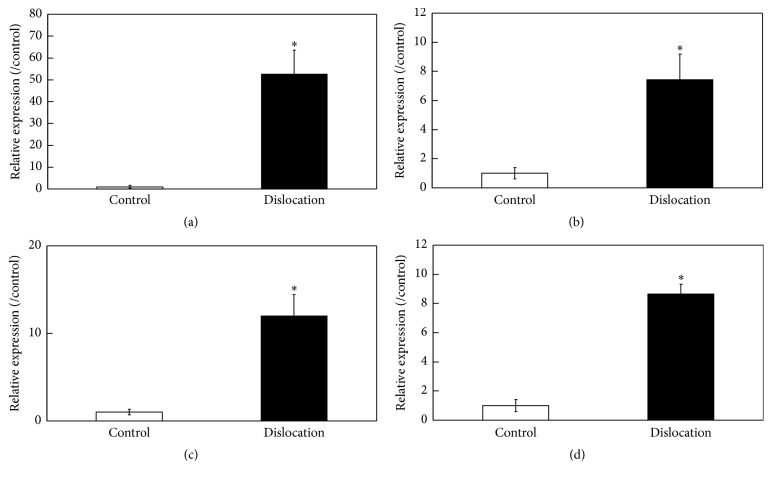
Real-time polymerase chain reaction (PCR) analysis of interleukin- (IL-) 1*β*, adrenomedullin (AM), receptor activity-modifying protein 2 (RAMP2), and RAMP3 gene expression in the synovial tissue of C57BL/6J mice. (a)* IL-1β*, (b) adrenomedullin* (AM)*, (c) receptor activity-modifying protein 2* (RAMP2)*, and (d)* RAMP3* gene expression in ST obtained from the control and dislocated side of a patellar surgical dislocation model generated in C57BL/6J mice. ^*∗*^Statistically significant difference between the control and dislocated side. All data are presented as the mean ± standard error (*n* = 5).

**Figure 3:**
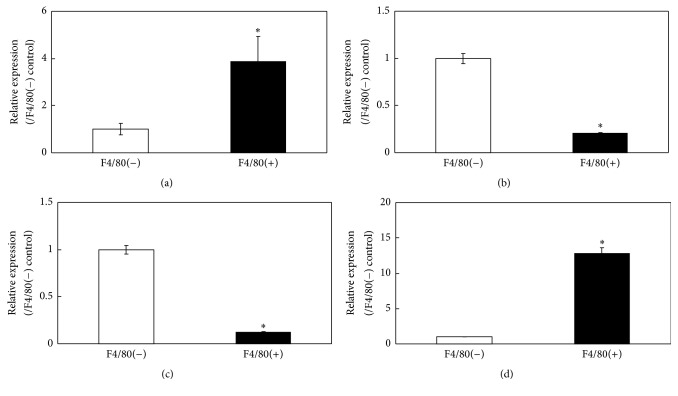
Real-time polymerase chain reaction (PCR) analysis for interleukin- (IL-) 1*β*, adrenomedullin (AM), receptor activity-modifying protein 2 (RAMP2), and RAMP3 gene expression in F4/80-negative and -positive cells derived from inflamed synovial tissue. (a)* IL-1β*, (b) adrenomedullin* (AM)*, (c) receptor activity-modifying protein 2* (RAMP2)*, and (d)* RAMP3* gene expression in ST obtained from the control and dislocated side of a patellar surgical dislocation model generated in C57BL/6J mice. ^*∗*^Statistically significant difference between F4/80-negative and F4/80-positive fractions. All data are presented as the mean ± standard error (*n* = 5).

Real-time PCR analysis of RNA extracted from the control and inflamed ST showed that the expression of the* CD11c*,* IL-1β*,* AM*,* RAMP2,* and* RAMP3* genes was significantly elevated in the ST from the dislocated side (Figures 2(a)–2(d)). In addition, expression of* IL-1β* and* RAMP3* in the F4/80+ fraction was significantly higher than that in F4/80− fraction (Figures 3(a) and 3(d)), whereas* AM* and* RAMP2 *expression was higher in the F4/80− fraction (Figures 3(b) and 3(c)).

### 3.3. Effect of Exogenous IL-1*β* on* F4/80*,* IL-1β*,* AM*,* RAMP2*, and* RAMP3* Gene Expression in Cultured Primary Synovial Fibroblasts and Macrophages

**Figure 4:**
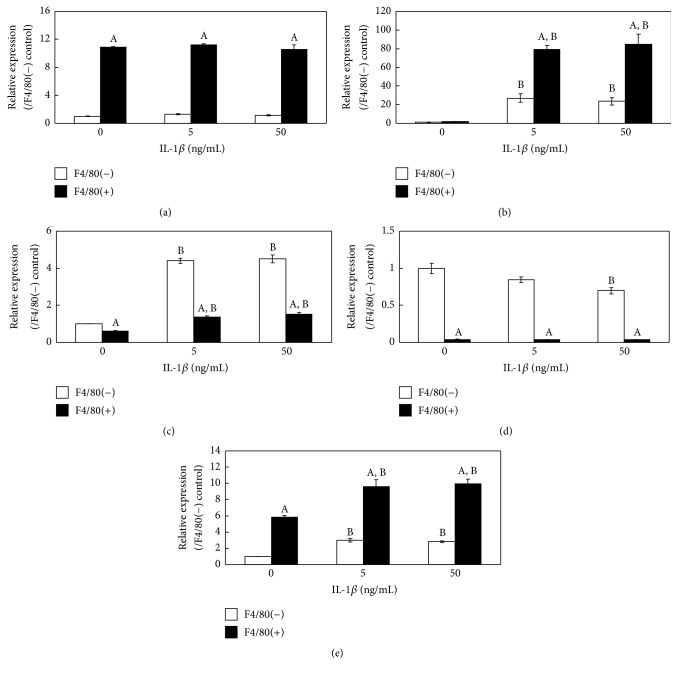
Effects of interleukin- (IL-) 1*β* on IL-1*β*, adrenomedullin (AM), and receptor activity-modifying protein 2 (RAMP2), and RAMP3 gene expression in cultured primary synovial fibroblasts and macrophages. The effects of IL-1*β* on* F4/80* (a),* IL-1β* (b),* AM* (c),* RAMP2* (d) and* RAMP3* (e) gene expression in cultured primary synovial fibroblasts (F4/80-negative fraction) and macrophages (F4/80-positive fraction) were examined by real-time PCR. ^A^*P* < 0.05 compared with F4/80-negative fraction. ^B^*P* < 0.05 compared with fraction matched-untreated controls.

The expression of the* F4/80*,* IL-1β*,* AM*,* RAMP2*, and* RAMP3* genes in cultured primary synovial fibroblasts and macrophages obtained from ST was measured before and after IL-1*β* stimulation. F4/80 expression in the F4/80+ fraction was significantly higher than that in F4/80− fraction and did not change by IL-1*β* stimulation in either fraction (Figure 4(a)). The expression of* IL-1β* and* RAMP3* increased significantly in both the F4/80− and F4/80+ cell fractions treated with exogenously added IL-1*β* compared to the untreated control cells (Figures 4(b) and 4(e)). In addition,* AM* and* RAMP2* expression in the F4/80− fraction was significantly higher than that in F4/80+ cell fraction, regardless of IL-1*β* stimulation. However,* AM *expression increased significantly in the F4/80− and F4/80+ cells fractions treated with IL-1*β* compared to the untreated control cells (Figures 4(c) and 4(d)).

### 3.4. Effects of AM on* IL-1β*,* AM*,* RAMP2*, and* RAMP3* Gene Expression in Synovial Fibroblasts and Macrophages

**Figure 5:**
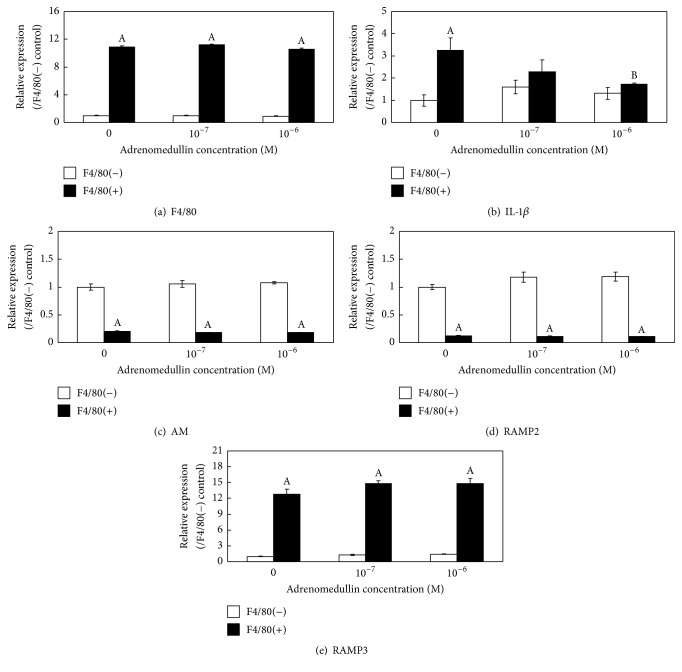
Effects of adrenomedullin (AM) on interleukin- (IL-) 1*β* gene expression in cultured primary synovial fibroblasts and macrophages. The effects of AM on* F4/80*,* IL-1β*,* AM*,* RAMP2*, and* RAMP3* gene expression in cultured primary synovial fibroblasts (F4/80-negative fraction) and macrophages (F4/80-positive fraction) were examined by real-time PCR. ^A^*P* < 0.05 compared with F4/80-negative fraction. ^B^*P* < 0.05 compared with fraction matched-untreated control.

F4/80 expression in the F4/80+ fraction was significantly higher than that in the F4/80− fraction and was not affected by AM stimulation of either fraction (Figure 5(a)).* IL-1β* expression in the F4/80+ cell fraction was significantly decreased in the presence of exogenously added AM (Figure 5(b)). In contrast,* AM*,* RAMP2*, and* RAMP3* gene expression in the F4/80− and F4/80+ cell fractions was not changed by treatment with AM (Figures 5(c)–5(e)).

## 4. Discussion

In the present study investigating the role of AM in synovial inflammation, CD11c^+^ populations of macrophages were found to be elevated in the ST of a murine patellar surgical dislocation model. Higher gene expression of the genes encoding* CD11c*,* IL-1β*,* AM*,* RAMP2*, and* RAMP3* was also observed in ST from the dislocated side compared to control ST. In addition, the expression of* AM* increased significantly in synovial fibroblasts and macrophages treated with exogenous IL-1*β*. Synovial macrophages were also found to highly express* RAMP3* compared to synovial fibroblasts, particularly when stimulated with exogenously added IL-1*β*. In addition, the treatment of ST-derived macrophages with AM inhibited* IL-1β* expression in the F4/80+ cell fraction. Taken together, these findings indicate that AM was produced by synovial fibroblasts and macrophages in response to synovial inflammation and that increased levels of AM may exert anti-inflammatory effects on synovial macrophages.

Synovitis contributes to the destruction of joint surfaces in osteoarthritis and rheumatoid arthritis [18, 19]. Chosa et al. found that plasma levels of AM in patients with rheumatoid arthritis (RA) are elevated compared to healthy individuals and that AM levels in synovial tissue and joint fluid in RA patients are significantly higher than those associated with osteoarthritis [20]. In the present study, the expression levels of AM and number of CD11c^+^ F4/80^+^ CD11b^+^ macrophages were significantly elevated in the ST obtained from the dislocated patella side of C57BL/6J mice. In addition, the ST macrophage fraction highly expressed* IL-1β*, which when exogenously added, stimulated AM expression in synovial cells. These findings are consistent the elevated IL-1*β* production previously observed in CD11c^+^ macrophages [16, 21] and the stimulation of AM production by IL-1*β* treatment in several cell types, including vascular smooth muscle cells and adipocytes [8, 22]. Taken together, the previous and present findings suggest that activated macrophages contribute to the stimulation of AM production in inflamed joints.

Vascular smooth muscle cells, endothelial cells, monocytes, and macrophages produce and secrete AM in vitro [14, 23–25]. Previous studies showed that AM is secreted by fibroblast-like synoviocytes in RA patients [26, 27]; however, the expression of* AM* in synovial macrophages was not elucidated. Here, the expression of* AM *increased significantly in a cell fraction consisting of synovial fibroblasts and macrophages in the presence of exogenously added IL-1*β*. However,* AM* gene expression in the F4/80− cell fraction was higher than that in the F4/80^+^ cell fraction. The present findings suggest that although synovial macrophages are a potential source for AM, synovial fibroblasts may be the major source of AM in inflamed synovial tissue.

AM binds to a heterodimeric plasma-membrane receptor composed of the calcitonin receptor-like receptor (CRLR) and receptor activity-modifying protein- (RAMP-) 2 or 3 [10, 11]. In the present study, the fibroblast cell fraction obtained from the inflamed ST of mice expressed significantly higher levels of* RAMP2* compared to the macrophage cell fraction, consistent with a report in which RAMP2 was expressed in the synovial fibroblasts of RA patients [28]. In contrast,* RAMP2* expression levels in macrophages were approximately 100-fold lower than that detected in synovial fibroblasts. In addition,* RAMP3* expression in synovial macrophages was markedly higher compared to that detected in fibroblasts and was also stimulated by exogenously added IL-1*β*. Uzan et al. also reported that RAMP3 protein was not detectable in synovial fibroblasts from RA patients [28]. The present findings suggest that AM acts on synovial macrophages through RAMP3 during synovial inflammation.

Several recent studies have provided evidence that AM has anti-inflammatory effects by regulating innate immunity responses, including the reduced production of proinflammatory cytokines, including TNF-alpha, IL-6, and IL-1 [14, 15, 29, 30]. For example, AM suppresses the in vitro secretion and gene transcription of TNF-*α* and IL-6 in the murine monocyte/macrophage cell line RAW 264.7 [14] and also attenuates TNF-*α* and IL-1*β* production in an in vivo acute lung injury mouse model [15]. In addition, subcutaneous AM injection suppresses the inflammation response in a collagen-induced arthritis mice model [31]. Intra-articular AM injection also reduces synovial inflammatory cytokine production in an antigen-induced arthritis rabbit model [32]. In the present study, expression of the gene encoding IL-1*β* was reduced in the AM-treated F4/80^+^ cell fraction obtained from inflamed ST. The present results suggest that AM and RAMP3 levels are increased in synovial macrophages in response to synovial inflammation and that AM may exert anti-inflammatory effects via the suppression of IL-1*β* production by macrophages.

Two limitations of the present study warrant mention. First, although F4/80 has been used as a murine macrophage marker [33, 34], a recent study indicated that F4/80 is required for the induction of efferent CD8+ regulatory T cells needed for peripheral tolerance [35]. In the present study, F4/80 was used for macrophage isolation, and it remains to be determined whether F4/80 expression contributes to AM/IL-1*β* cross-talk. Second, the obtained research data cannot be directly extrapolated to human subjects because human EMR1 is a marker of eosinophils but is not expressed on macrophages.

In conclusion, AM production was increased in synovial fibroblasts and macrophages in a murine patellar surgical dislocation model. In inflamed ST, AM appears to mediate anti-inflammatory effects by inhibiting IL-1*β* secretion by synovial macrophages. Adrenomedullin may constitute a key target in future therapies for inflammatory arthritis.

## Figures and Tables

**Table 1:** Sequences of the primers used in this study.

Gene	Direction	Primer sequence (5′-3′)	Product size (bp)
*CD11c*	F	TTCTTCTGCTGTTGGGGTTTG	132
	R	CAACCACCACCCAGGAACTAT	
*IL-1β*	F	GCAACTGTTCCTGAACTCAACT	89
	R	ATCTTTTGGGGTCCGTCAACT	
*AM*	F	TAAGTGGGCGCTAAGTCGTG	74
	R	TCTCATCAGCGAGTCCCGTA	
*RAMP2*	F	CACTGAGGACAGCCTTGTGT	117
	R	GTCCAGTTGCACCAGTCCTT	
*RAMP3*	F	CTGCAACGAGACAGGGATGC	95
	R	GGTTGCACCACTTCCAGACA	
*F4/80*	F	TGGGATGTACAGATGGGGGA	189
	R	CCTGGGCCTTGAAAGTTGGT	
*GAPDH*	F	AACTTTGGCATTGTGGAAGG	223
	R	ACACATTGGGGGTAGGAACA	
